# The Folding Pathway of a Single Domain in a Multidomain Protein is not Affected by Its Neighbouring Domain

**DOI:** 10.1016/j.jmb.2008.02.032

**Published:** 2008-04-25

**Authors:** Sarah Batey, Jane Clarke

**Affiliations:** Cambridge University Chemical Laboratory, MRC Centre for Protein Engineering, Lensfield Road, Cambridge CB2 1EW, UK

**Keywords:** R15, the 15th α-helical repeat from chicken brain α-spectrin, R16, the 16th α-helical repeat from chicken brain α-spectrin, R1516, the 15th and 16th α-helical repeats from chicken brain α-spectrin in a tandem construct, spectrin, domain–domain interactions, cooperativity, multidomain proteins, protein folding mechanisms

## Abstract

Domains are the structural, functional, and evolutionary components of proteins. Most folding studies to date have concentrated on the folding of single domains, but more than 70% of human proteins contain more than one domain, and interdomain interactions can affect both the stability and the folding kinetics. Whether the folding pathway is altered by interdomain interactions is not yet known. Here we investigated the effect of a folded neighbouring domain on the folding pathway of spectrin R16 (the 16th α-helical repeat from chicken brain α-spectrin) by using the two-domain construct R1516. The R16 folds faster and unfolds more slowly in the presence of its folded neighbour R15 (the 15th α-helical repeat from chicken brain α-spectrin). An extensive Φ-value analysis of the R16 domain in R1516 was completed to compare the transition state of the R16 domain alone with that of the R16 domain in a multidomain construct. The results indicate that the folding pathways are the same. This result validates the current approach of breaking up larger proteins into domains for the study of protein folding pathways.

A protein domain is a structural, functional, and evolutionary component of proteins. Analysis of genomic sequence data has shown that at least two-thirds of all eukaryotic proteins contain more than one domain.^1^ Domain shuffling has resulted in the evolution of new proteins with new functionalities; thus, the assumption is that each domain is an autonomous folding unit. The corollary of this is the assumption that studies of the folding of small isolated domains can be applied to the same domains in larger multidomain proteins. Only a small number of studies on the thermodynamic and kinetic properties of multidomain proteins have been carried out.^2–12^ In some cases, domains appear to be entirely independent of their neighbours; in others, domains are stabilised by interdomain interactions. A recent analysis of multidomain protein folding studies investigated the relationship between the interface structure as well as packing density and the interdependence of the folding of domains in multidomain proteins.^13^ This analysis suggested that the domains will fold independently where they have a flexible linker and a small interface; that is, the equilibrium and kinetic properties will be the same whether they are studied as a single domain or as part of a multidomain protein. Proteins with a large interface appear to be coupled to some degree. In most cases, the effect simply is stabilisation of the final folded native state, presumably through favourable interface interactions, such that the stabilisation results only in a decrease in the unfolding rate constant. In only two of the multidomain proteins studied were both folding and unfolding rate constants affected by interdomain interactions.^2,3,6^ One of these cases is the extensively studied protein spectrin, which comprises multiple three-helix bundle domains each joined by a helix that extends from one domain to another (Fig. 1a). The folding of spectrin domains is accelerated by the presence of a folded domain at the N-terminus. We used this as a model protein system to investigate whether the folding pathway itself is perturbed in such a case.

Previous studies on domain pairs R1516 (the 15th and 16th α-helical repeats from chicken brain α-spectrin in a tandem construct) and R1617 had shown that, in both cases, each domain is stabilised by specific interactions with its neighbour,^2–4,15–17^ and the folding pathways of the isolated domains have been elucidated^18–20^ (and unpublished data). For R1516, the first step in the pathway is the folding of the N-terminal R15 domain (the 15th α-helical repeat from chicken brain α-spectrin) that results in the formation of an intermediate with a folded R15 domain and an unfolded R16 domain (the 16th α-helical repeat from chicken brain α-spectrin) (Fig. 1b).^2^ This first step is rapid (folding rate constant in water, *k*_f_^H2O^ ≈ 30,000 s^− 1^). The next step is the folding of R16 in the presence of folded R15. The presence of this neighbouring folded domain results in a sixfold increase in the *k*_f_^H2O^ of R16 (∼ 750 *versus* 125 s^− 1^ for R16 alone). The unfolding pathway is the reverse of the folding pathway. R16 unfolds first, resulting in an intermediate with R15 folded and R16 unfolded. R16 unfolds more slowly in the presence of folded R15 (*k*_u_^H2O^ = 7.5 × 10^− 4^ *versus* 2.6 × 10^− 3^ for R16 alone). The unfolding of R16 results in the loss of stabilisation of R15, leading to the rapid unfolding of R15.

In summary, the R16 domain folds faster and unfolds more slowly when attached to a folded R15 domain than it does when on its own. Thus, the spectrin R1516 system is an ideal candidate to investigate whether the interdomain interactions, which promote folding and slow unfolding, alter the folding pathway.

The folding pathway of R16 has been extensively characterised by protein engineering Φ-value analysis.^19^ Two types of mutation were made to determine the folding pathway of R16 in R1516: conservative core deletion mutations, to probe the formation of tertiary contacts, and solvent-exposed (Ala → Gly) mutations, to probe the formation of the secondary structure. The same sites as those in the previous R16 study were chosen to allow direct comparison of the folding pathway of R16 in R1516 with that of R16 alone. The kinetics of the mutant proteins were followed by the change in fluorescence, as previously described.^4^ The R15 domain was not affected by any of the mutations we made in the R16 domain of R1516. Examples of chevron plots obtained are shown in Fig. 2; all chevron plots comparing the R16 domain in the R1516 mutants with the R16 domain in wild-type R1516 are shown in Supplementary Fig. S1.

The phases due to the folding and unfolding kinetics of R16 were fit to a sequential transition state model (Supplementary Fig. S2).^19,21–23^ In this model, which accounts for the curvature observed in the unfolding arm, there is a switch between transition states separated by a high-energy intermediate. At low concentrations of denaturant, the less structured transition state (TS_early_) is rate limiting; at high concentrations of denaturant, the more structured transition state (TS_late_) becomes rate limiting. However, as many mutants do not reach a limiting slope in the unfolding limb in the experimental range of denaturant concentrations, it was necessary to fit the data globally to determine this limiting slope, as was described for isolated R16.^19^ The results of the fits are given in Supplementary Table S1.

The change in free energy upon mutation (ΔΔ*G*_D–N_) of R16 in R1516 cannot be determined by equilibrium measurements due to overlapping of the denaturation curves of the individual R15 and R16 domains.^2^ Since the error of the Φ-value is largely determined by the error in ΔΔ*G*_D–N_, it is important to have accurate values for ΔΔ*G*_D–N_. We plotted the kinetically determined ΔΔ*G* values from the R16 domain of R1516 against those of the individual R16 equilibrium measurements (Supplementary Fig. S3). The plot shows a strong linear dependence with a slope of close to 1. This indicates that the ΔΔ*G* values determined from the equilibrium denaturation of R16 alone are a good representation of the ΔΔ*G* of R16 in R1516. The equilibrium values were used in the calculation of the Φ-values due to smaller errors associated; however, when the kinetic values from R1516 were used, the Φ-value patterns were essentially the same.

From the fit of the data to a sequential transition state model,^23^ it was possible to determine Φ-values for both the early and late transition states of R16 in R1516. Note that the Φ-values for TS_late_ are associated with large errors,^19,21^ especially in R1516, where the unfolding limbs are shorter and there are too few data points to determine the folding and unfolding rate constants associated with TS_late_ accurately.

The Φ-values from individual R16 and R16 within R1516 are shown in Fig. 3 and Supplementary Table S1. The patterns of Φ-values in R16 alone and in R16 in R1516 are clearly the same. For TS_early_, which is rate limiting at 0 M denaturant, the mean Φ-values are the same (0.25 and 0.26 for R16 alone and that in R1516, respectively) and the patterns are the same (significant helical structure in helix C with a lesser degree of secondary and tertiary structure in helix A and little structure formation in helix B). In TS_late_, which is rate limiting at high denaturant concentrations, the Φ-values are uniformly higher in R16 in R1516 than in R16 alone (mean Φ-values of 0.66 and 0.52, respectively), but, again, the patterns are the same. There is a concomitant increase in secondary and tertiary structures in helix A, and the tertiary structure in helix C increases; there is also evidence (as with R16) of the N-terminal region of helix B forming strong tertiary contacts with the C-terminal region of helix C. Thus, the folding pathway of R16 is apparently unchanged by attachment of a neighbouring folded R15 domain.^3,4,17^

How then can we explain the higher folding rate constant of R16 in R1516 compared with R16 alone? It is always possible in multidomain proteins that changes in kinetics are due to intermolecular “crowding effects,” since each domain is in close association with its neighbour.^24^ However, we have recently ruled out this possibility. We have shown that the folding rate constant of R16 is unaffected by the presence of a neighbouring folded nonnative domain at the N-terminus in an artificial two-domain protein construct of titin I27–R16.^17^ Furthermore, the folding of R16 is not accelerated by the presence of an unfolded R17 domain at the C-terminus. Thus, the acceleration of the folding of R16 by folded R15 appears to be a specific event. Note that the same principles are true for the homologous R1617 pair. The folding of R17 is only accelerated by a fully folded R16 domain at the N-terminus; folding is not accelerated by an unfolded R16 domain, by a fragment of the R16 domain, or by a nonnatural folded titin domain at the N-terminus.^3,4,17^

There is evidence that the denatured state of R16 in R1516 is more compact than when R16 is studied alone. It has a consistently lower *m*_D–N_ than R16 alone for all mutations (average *m*-values of 1.7 ± 0.1 and 1.9 ± 0.1 kcal mol^− 1^ M^− 1^, respectively). The globally fitted unfolding *m*-values, related to the change in solvent-accessible surface area between the native state and the two transition states, are exactly the same as for the unfolding of R16 and all its mutants alone,^19^ indicating that the position of the transition states of R16 along the reaction coordinate, relative to the native state, remains the same whether or not the domain has a folded neighbour. The folding *m*-value (*m*_kf_) for R16 in R1516 is, however, significantly lower than that for the same mutants in R16 alone (average *m*_D–TS_ values of 1.0 ± 0.1 and 1.2 ± 0.1 kcal mol^− 1^ M^− 1^, respectively). Thus, the change in *m*_D–N_ is accounted for entirely by the lowering of the refolding *m*-value (i.e., the solvent-accessible surface area of the denatured state is closer to that of the TS_early_ in R1516).

R16 in R1516 is folding in the presence of folded R15, and the linker between the two is an extended helix (Fig. 1a). If the preformed structure of helix C of R15 is extending into helix A of R16 in the denatured state, this could explain the decrease in *m*_kf_ and in *m*_D–N_. Three additional exposed sites, which had not been previously studied (K1, S5, and H6, all mutated to both Ala and Gly), were mutated in R16 and R1516 to investigate the possibility of helical structure in the early part of helix A of R16. However, none of these mutations had any significant effect on ΔΔ*G*_D–N_ in either R16 alone or R16 in R1516, suggesting that there is no significant structure in this region in either construct. This also means that Φ-values could not be calculated, although visual inspection of the chevrons does not suggest that the Φ-values in this region are large. Possibly, the increased compactness in the denatured state simply reduces the entropic cost of folding and results in the faster folding of R16.

The study of multidomain proteins is important in bridging the gap in the knowledge of protein folding between single domains and the same domains in their natural molecular environment. Here we show for the first time that the Anfinsen principle holds for single domains within a multidomain protein system. Although interdomain interactions may stabilise native structures and, more rarely, partly folded transition state structures, thereby both slowing unfolding and/or speeding folding, the folding pathway is prescribed by the primary sequence of the single domain alone. Thus, evolution, through domain shuffling, does not apparently require reengineering of protein folding energy landscapes. This lends support to the existing approach of studying individual domains from a multidomain protein in isolation.

## Figures and Tables

**Fig. 1:**
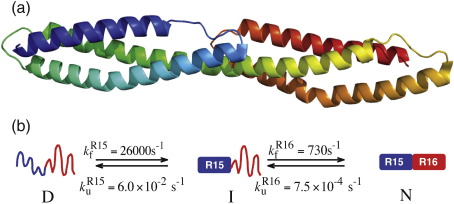
The structure and folding pathway of spectrin R1516. (a) R1516 is a two-domain fragment from chicken brain α-spectrin (from Protein Data Bank file 1U4Q).^14^ The chain is shown from blue (A-helix of R15) to red (C-helix of R16). The C-helix of R15 forms a continuous helix with the A-helix of R16. (b) Folding pathway of R1516 adapted from Ref.^2^. The R15 domain folds rapidly to form a stable intermediate with R15 folded and R16 unfolded. The R16 domain folds more slowly than R15. In the unfolding direction, R16 unfolds first. Loss of interactions with R16 destabilises the R15 domain such that this unfolds rapidly.

**Fig. 2:**
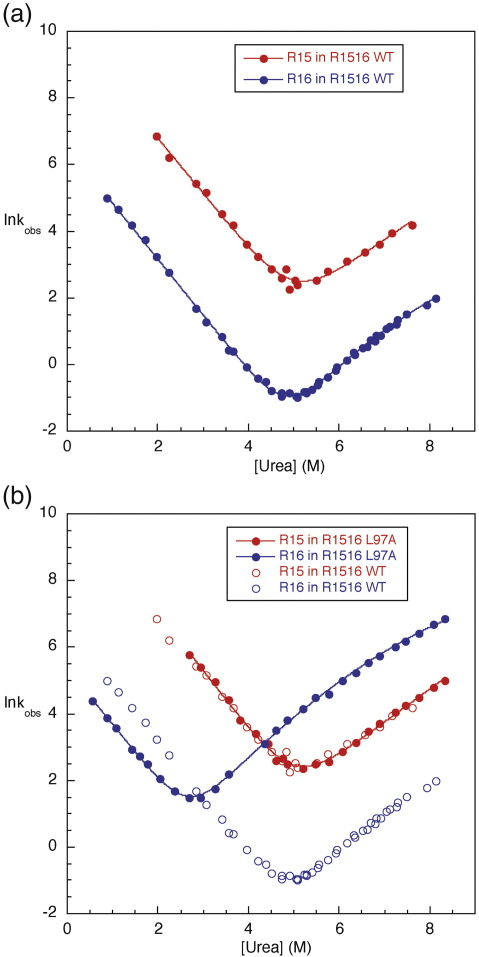
Chevron plots of wild type (WT) R1516 and a mutant. The proteins were all purified as previously described.^3^ The kinetics were analysed as previously described.^2,4^ (a) For WT R1516, there are three observable folding phases and one observable unfolding phase. The fastest folding phase has approximately 30% of the total amplitude, the intermediate phase has approximately 60%, and the slowest of the three phases (not shown here) has less than 10%. The fast refolding phase has been attributed to the folding of R15 (red closed circles); the middle refolding phase, to the folding of R16 (blue closed circles); and the slowest of the phases, to prolyl isomerisation in WT R1516. The single observable unfolding phase is the unfolding of R16. R15 can be seen to unfold in double-jump experiments. Data were taken from Ref. 2. (b) Chevron plot for the mutant L97A in the R16 domain of R1516 compared with WT R1516. The unfolding of the R15 domain is unaffected by any of the mutations (shown in red: closed circles, mutant; open circles, WT). The L97A mutation slows the unfolding of the R16 domain slightly but speeds the unfolding of the R16 domain significantly (i.e., the Φ-value is low, 0.3) (shown in blue: closed circles, mutant; open circles, WT). In this case, the R16 domain unfolds so rapidly that both unfolding phases can be observed in a single-jump experiment. In Supplementary Data, chevron plots for the R16 domain in R1516 are shown for all mutant proteins, compared with R16 in WT R1516.

**Fig. 3:**
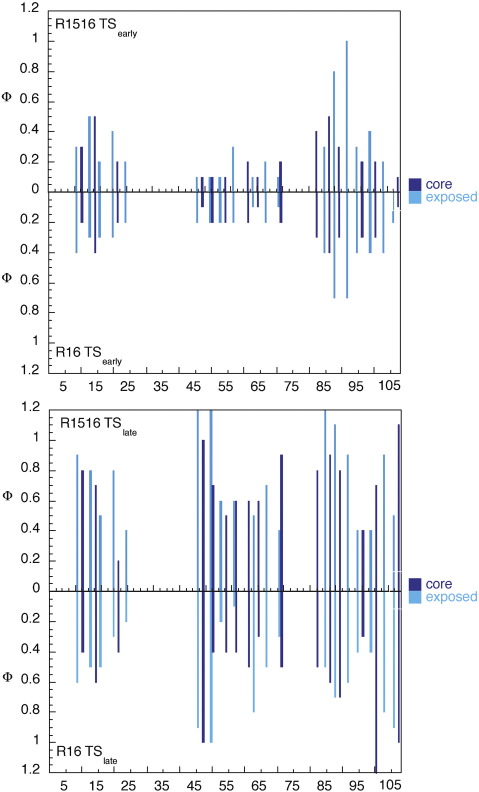
Comparison of the Φ-values for R16 alone and in R1516. The patterns of the Φ-values are essentially the same whether the R16 domain is in isolation or attached to folded R15. The darker bars are the Φ-values for surface Ala → Gly mutants, which probe secondary structure formation. The lighter bars are for mutations of buried residues to Ala, which probe the tertiary structure. In the early rate-determining transition state (top panel), the magnitude and the patterns of Φ-values are the same. In the late transition state (lower panel), which is only rate determining at high denaturant concentrations, the Φ-values are slightly higher for the R16 domain in R1516, but, again, the patterns of Φ-values are the same. Note that the error in Φ_late_ is relatively high, especially for R16 in R1516, due to uncertainties in fitting the data where there is little curvature in the unfolding arm (see Refs. 19,21 and the text). This is likely why there are some nonclassical Φ-values (> 1). Data for R16 alone were taken from Ref. 19.

## References

[1] Teichmann S.A., Chothia C., Gerstein M. (1999). Advances in structural genomics. Curr. Opin. Struct. Biol..

[2] Batey S., Clarke J. (2006). Apparent cooperativity in the folding of multidomain proteins depends on the relative rates of folding of the constituent domains. Proc. Natl Acad. Sci. USA.

[3] Batey S., Randles L.G., Steward A., Clarke J. (2005). Cooperative folding in a multi-domain protein. J. Mol. Biol..

[4] Batey S., Scott K.A., Clarke J. (2006). Complex folding kinetics of a multidomain protein. Biophys. J..

[5] Jager M., Gehrig P., Pluckthun A. (2001). The scFv fragment of the antibody hu4D5–8: evidence for early premature domain interaction in refolding. J. Mol. Biol..

[6] Osvath S., Kohler G., Zavodszky P., Fidy J. (2005). Asymmetric effect of domain interactions on the kinetics of folding in yeast phosphoglycerate kinase. Protein Sci..

[7] Politou A.S., Gautel M., Improta S., Vangelista L., Pastore A. (1996). The elastic I-band region of titin is assembled in a “modular” fashion by weakly interacting Ig-like domains. J. Mol. Biol..

[8] Robertsson J., Petzold K., Lofvenberg L., Backman L. (2005). Folding of spectrin's SH3 domain in the presence of spectrin repeats. Cell. Mol. Biol. Lett..

[9] Rothlisberger D., Honegger A., Pluckthun A. (2005). Domain interactions in the Fab fragment: a comparative evaluation of the single-chain Fv and Fab format engineered with variable domains of different stability. J. Mol. Biol..

[10] Scott K.A., Steward A., Fowler S.B., Clarke J. (2002). Titin; a multidomain protein that behaves as the sum of its parts. J. Mol. Biol..

[11] Steward A., Adhya S., Clarke J. (2002). Sequence conservation in Ig-like domains: the role of highly conserved proline residues in the fibronectin type III superfamily. J. Mol. Biol..

[12] Wenk M., Jaenicke R., Mayr E.M. (1998). Kinetic stabilisation of a modular protein by domain interactions. FEBS Lett..

[13] Han J.H., Batey S., Nickson A.A., Teichmann S.A., Clarke J. (2007). The folding and evolution of multidomain proteins. Nat. Rev. Mol. Cell Biol..

[14] Kusunoki H., Minasov G., Macdonald R.I., Mondragon A. (2004). Independent movement, dimerization and stability of tandem repeats of chicken brain alpha-spectrin. J. Mol. Biol..

[15] Macdonald R.I., Pozharski E.V. (2001). Free energies of urea and of thermal unfolding show that two tandem repeats of spectrin are thermodynamically more stable than a single repeat. Biochemistry.

[16] MacDonald R.I., Cummings J.A. (2004). Stabilities of folding of clustered, two-repeat fragments of spectrin reveal a potential hinge in the human erythroid spectrin tetramer. Proc. Natl Acad. Sci. USA.

[17] Randles L.G., Batey S., Steward A., Clarke J. (2008). Distinguishing specific and non-specific inter-domain interactions in multidomain proteins. Biophys. J..

[18] Scott K.A., Batey S., Hooton K.A., Clarke J. (2004). The folding of spectrin domains: I. Wild-type domains have the same stability but very different kinetic properties. J. Mol. Biol..

[19] Scott K.A., Randles L.G., Clarke J. (2004). The folding of spectrin domains: II. Phi-value analysis of R16. J. Mol. Biol..

[20] Scott K.A., Randles L.G., Moran S.J., Daggett V., Clarke J. (2006). The folding pathway of spectrin R17 from experiment and simulation: using experimentally validated MD simulations to characterize states hinted at by experiment. J. Mol. Biol..

[21] Scott K.A., Clarke J. (2005). Spectrin R16: broad energy barrier or sequential transition states?. Protein Sci..

[22] Sanchez I.E., Kiefhaber T. (2003). Evidence for sequential barriers and obligatory intermediates in apparent two-state protein folding. J. Mol. Biol..

[23] Bachmann A., Kiefhaber T. (2001). Apparent two-state tendamistat folding is a sequential process along a defined route. J. Mol. Biol..

[24] Minton A.P. (2000). Implications of macromolecular crowding for protein assembly. Curr. Opin. Struct. Biol..

