# Supplementary material for: The Folding Pathway of a Single Domain in a Multidomain Protein is not Affected by Its Neighbouring Domain
Source: J Mol Biol. 2008 Apr 25;378(2):297–301. doi: 10.1016/j.jmb.2008.02.032 (PMC2828540; doi:10.1016/j.jmb.2008.02.032)
Supplement: Supplementary material 1 [file mmc1.pdf]

Supplementary Data

Table S1 Equilibrium and kinetic parameters for R16 mutants

Mutant[a]	$\Delta G_{(kin)}$ (kcal mol^{-1}) Determined in R1516[b]	$\Delta\Delta G_{(kin)}$ (kcal mol^{-1}) Determined in R1516	$\Delta\Delta G_{(eq)}$ (kcal mol^{-1}) Determined in isolated R16[c]	$k_f^{H_2O}$ (s^{-1}) (early)	$k_f^{H_2O}$ (s^{-1}) (late)	Φ^{R1516}_{early}	Φ^{R1516}_{late}	$\Phi^{R16\ c}_{early}$	$\Phi^{R16\ c}_{late}$
WT	8.2	-	-	725	390,000	-	-	-	-
K1A	7.2	-		200	146,000				
K1G	7.6	-0.4		470	207,000				
S5A	7.6	-		510	109,000				
S5G	8.0	-0.4		820	2370,000				
H6A	8.0			730	216,000				
H6G	7.9	0.1		630	761,000				
H9A	7.8	-	-	450	363,000	-	-	-	-
H9G	7.2	0.6	1.1	260	720,000	0.3	0.9	0.4	0.6
F11A	4.6	3.6	3.6	100	2,930	0.3	0.8	0.2	0.4
R13A	7.6	-	-	700	67,800	-	-	-	-
R13G	6.3	1.3	1.6	210	8,060	0.5	0.8	0.3	0.5
M15A	6.4	1.8	2.1	120	27,800	0.5	0.7	0.4	0.6
D16A	7.7	-	-	520	152,000	-	-	-	-
D16G	7.3	0.4	0.9	370	70,800	0.2	0.5	0.3	0.5
S20A	8.5	-	-	910	610,000	-	-	-	-
S20G	7.6	0.9	1.0	450	152,000	0.4	0.8	0.3	0.3
I22A	4.9	3.3	3.5	250	125,000	0.2	0.2	0.2	0.4
E24A	8.1	-	-	740	387,000	-	-	-	-
E24G	7.4	0.7	1.2	530	184,000	0.2	0.4	0.2	0.2

K46A	8.6	-	-	750	689,000	-	-	-	-
K46G	7.5	1.1	0.9	590	62,300	0.1	1.5	0.2	0.9
H48A	7.0	1.2	1.4	580	33,000	0.1	1.0	0.1	1.0
R50A	8.7	-	-	890	501,000	-	-	-	-
R50G	7.5	1.2	0.9	800	55,500	0.1	1.4	0.2	1.0
L51A	5.3	2.9	2.7	450	17,000	0.1	0.7	0.2	0.4
A53G	7.0	1.2	1.1	660	121,000	0.1	0.6	0.2	0.2
L55A	4.0	4.2	3.7	390	17,600	0.1	0.5	0.2	0.4
A57G	6.9	1.3	1.0	425	136,000	0.3	0.6	0.2	0.1
H58A	6.2	2.0	2.3	600	42,600	0.0	0.6	0.0	0.4
I62A	5.4	2.8	2.8	340	21,700	0.2	0.6	0.2	0.5
Q63A	7.4	-	-	765	101,000	-	-	-	-
Q63G	6.6	0.8	1.1	580	41,300	0.1	0.5	0.1	0.8
V65A	6.1	2.1	2.3	460	35,000	0.1	0.6	0.1	0.3
D67A	8.1	-	-	860	348,000	-	-	-	-
D67G	7.2	0.9	1.3	530	83,100	0.2	0.7	0.2	0.5
K71A	8.0	-	-	760	209,000	-	-	-	-
K71G	7.4	0.6	1.3	615	82,600	0.1	0.4	0.1	0.3
L72A	5.7	2.5	2.3	310	13,200	0.2	0.9	0.2	0.5
I83A	6.5	1.7	2.0	200	23,100	0.4	0.8	0.3	0.5
Q85A	8.2	-	-	890	343,000	-	-	-	-
Q85G	6.8	1.4	1.3	495	26,000	0.3	1.2	0.4	0.5
L87A	5.8	2.4	2.7	75	7,310	0.5	0.9	0.4	0.6
A88G	7.2	1.0	1.0	200	60,900	0.8	1.1	0.7	0.7
F90A	5.9	2.3	2.7	180	10,200	0.3	0.8	0.3	0.7
D92A	8.8	-	-	570	144,000	-	-	-	-
D92G	7.7	1.1	1.0	3500	877,000	1.0	0.9	0.7	0.6
K95A	7.6	-	-	350	136,000	-	-	-	-
K95G	6.9	0.7	0.9	220	75,700	0.3	0.4	0.4	0.4
L97A	4.4	3.8	3.7	250	38,700	0.2	0.4	0.3	0.3

Q99A	8.0	-	-	970	152,000	-	-	-	-
Q99G	7.1	0.9	1.1	470	84,100	0.4	0.4	0.4	0.4
A101G	5.5	2.7	2.6	370	17,900	0.2	0.7	0.3	0.5[d]
A103G	7.0	1.2	1.7	390	29,200	0.2	0.9	0.4	0.8
Q106A	8.2	-	-	720	218,000	-	-	-	-
Q106G	7.5	0.7	1.7	770	48,000	0.0	0.5	0.2	0.9
L108A	6.9	1.3	1.4	530	28,700	0.1	1.1	0.1	1.0

For clarity, errors are not shown in the Table. The approximate errors in free energies of unfolding are as follows: $\Delta G_{(kin)}$, ± 0.2 kcal mol^{-1}; $\Delta\Delta G_{(kin)}$, ± 0.3 kcal mol^{-1}; $\Delta\Delta G_{(eq)}$, ± 0.1 – 0.2 kcal mol^{-1}. The errors in the kinetic measurements are approximately: $k_f^{H_2O}$(early), < 10%; ; $k_f^{H_2O}$(late) < 25%. The errors in Φ-values are; Φ_{early}, ~0.1; Φ_{late}, ~0.2 – 0.3.

a For composite surface, helix-scanning mutations the $\Delta\Delta G$ and Φ-values reported are for the composite Ala → Gly mutation
b $\Delta G_{(kin)}$ for R1516 determined from the folding and unfolding rate constants (TS_{early}), extrapolated to 0 M denaturant, determined from the fit of the entire chevron plot as described.
c The $\Delta G_{(eq)}$ data (taken from equilibrium experiments on R16 alone) and the Φ-values for R16 alone are taken from Scott, K.A., Randles, L.G. & Clarke, J. (2004) The folding of spectrin domains II. Phi-value analysis of R16. *J. Mol. Biol.* **344**, 207–221
d Note that a typographical error in Scott *et al*. caused us to misquote the Φ-value for TS_{late} of A101G to be 1.5 instead of 0.5 in R16.
