# Supplementary material for: The Folding Pathway of a Single Domain in a Multidomain Protein is not Affected by Its Neighbouring Domain
Source: J Mol Biol. 2008 Apr 25;378(2):297–301. doi: 10.1016/j.jmb.2008.02.032 (PMC2828540; doi:10.1016/j.jmb.2008.02.032)
Supplement: Supplementary material 2 [file mmc2.pdf]

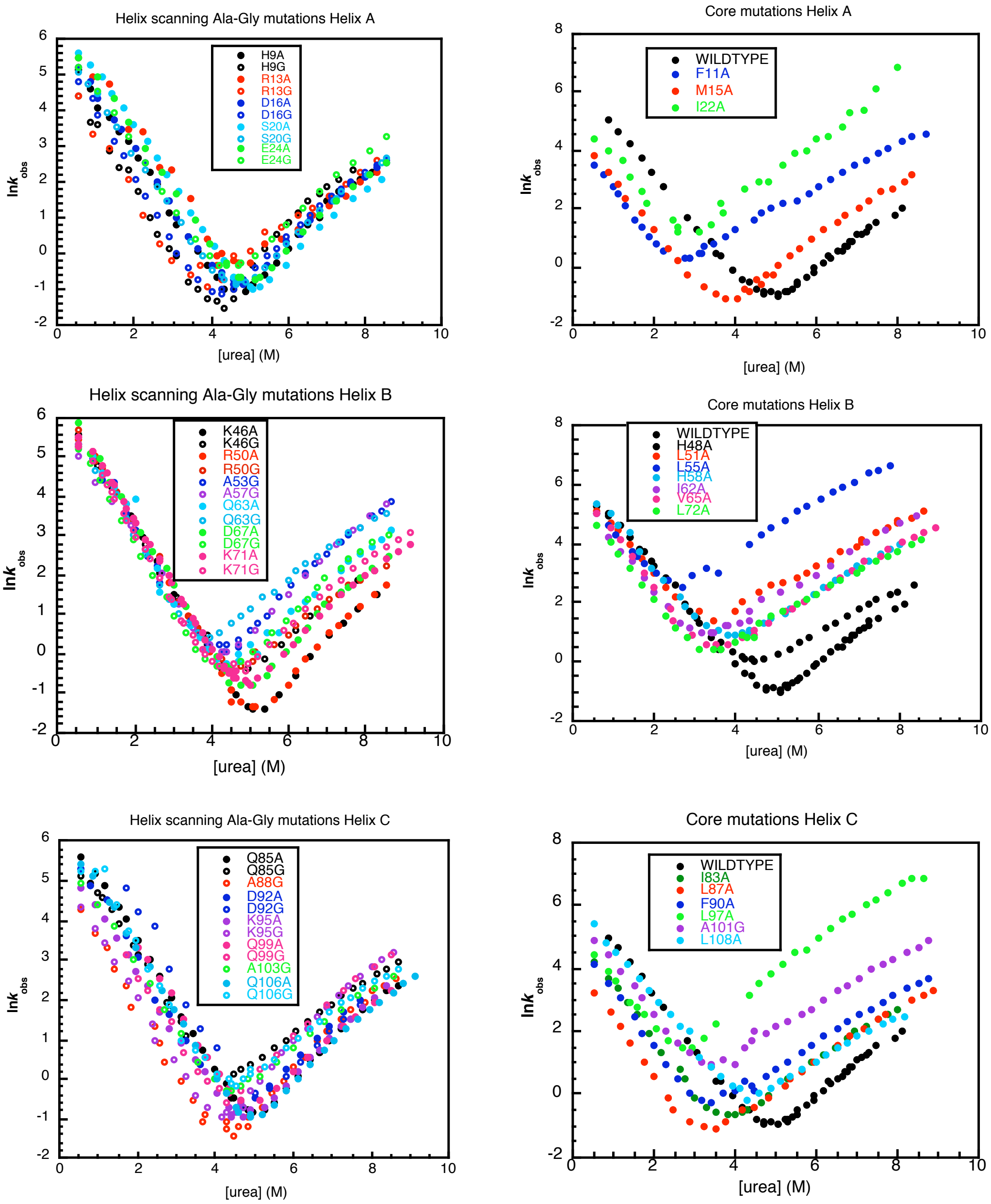

Figure S1. Chevron plots for all the mutant forms of the R16 domain in R1516. The mutants have been divided in helix-scanning Ala → Gly mutants (left hand column), which probe secondary structure, and core mutants (right hand column). Top row, A helix; middle row, B helix; bottom row C helix.

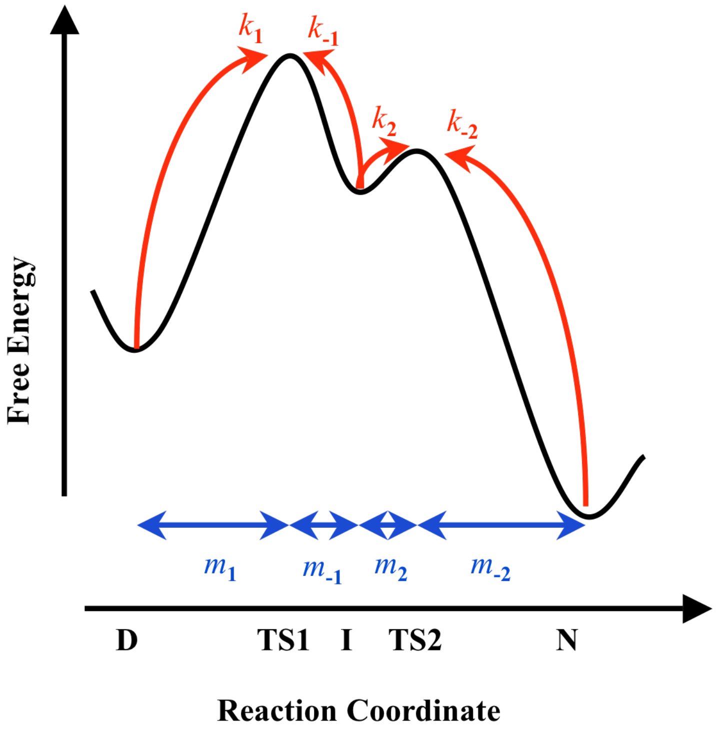

Figure S2. The high energy intermediate model used to fit the kinetic data. The equations used to fit data to this model are described in detail elsewhere[1-3].
The data for all mutants were fit globally with shared values of m_{-2} and m_2, but with m_1 allowed to float. In fitting this model m_{-1} is fixed to zero so that m_{-2} is actually a measure of ($m_{-1} + m_{-2}$). k_{-1} is arbitrarily fixed to 1×10^5, so that the stability of the intermediate can be determined ($K_I = k_{-1}/k_2$) but k_2 is only determined in relation to k_{-1}.
k_1 is the folding rate constant over the rate limiting TS1 (TS_{early}). The rate constant for unfolding over the same rate limiting TS1 is ($k_{-1}.k_{-1}/k_2$).
k_2 is the rate constant for unfolding over TS2 (the transition state that is only rate determining at high denaturant concentrations, TS_{late}). The rate constant for folding over TS2 is ($k_1.k_2/k_{-2}$).
The m-value $m_{D\text{-}N} = RT(m_1+m_{-1}+m_2+m_{-2})$. (Units of $m_{D\text{-}N}$, kcal mol^{-1} M^{-1}, units of kinetic m-values, M^{-1})

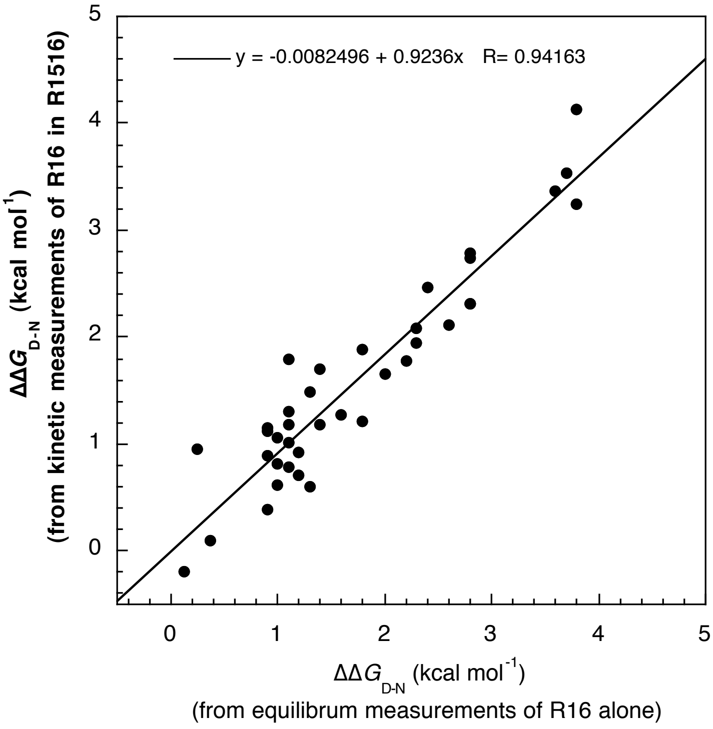

Figure S3. Comparison of $\Delta\Delta G_{\text{D-N}}$ determined using equilibrium denaturant experiments on R16 alone with the kinetic $\Delta\Delta G_{\text{D-N}}$ determined for the R16 domain in R1516. There is good agreement between the two sets of figures, but as the $\Delta\Delta G_{\text{D-N}}$ from the equilibrium data are associated with lower error these data are used to determine Φ-values. However, the pattern of Φ-values determined using the kinetic $\Delta\Delta G_{\text{D-N}}$ values is the same (data not shown). Data for R16 alone taken from[3].

1. Sanchez, I. E. & Kiefhaber, T. (2003). Evidence for sequential barriers and obligatory intermediates in apparent two-state protein folding. *J. Mol. Biol.* **325**, 367-376.
2. Bachmann, A. & Kiefhaber, T. (2001). Apparent two-state tendamistat folding is a sequential process along a defined route. *J. Mol. Biol.* **306**, 375-386.
3. Scott, K. A., Randles, L. G. & Clarke, J. (2004). The folding of spectrin domains II: phi-value analysis of R16. *J. Mol. Biol.* **344**, 207-221.
